# Defect Width Assessment Based on the Near-Field Magnetic Flux Leakage Method

**DOI:** 10.3390/s21165424

**Published:** 2021-08-11

**Authors:** Erlong Li, Yiming Chen, Xiaotian Chen, Jianbo Wu

**Affiliations:** 1 School of Mechanical Engineering, Sichuan University, Chengdu 610065, China; lierlg@scu.edu.cn (E.L.); 2017141414029@stu.scu.edu.cn (Y.C.); wujianbo@scu.edu.cn (J.W.); 2 School of Electrical & Electronic Engineering, Newcastle University, Newcastle upon Tyne NE1 7RU, UK

**Keywords:** magnetic flux leakage (MFL), near-field effect, magnetic dipole, quantitative assessment

## Abstract

Magnetic flux leakage (MFL) testing has been widely used as a non-destructive testing method for various materials. However, it is difficult to separate the influences of the defect geometrical parameters such as depth, width, and length on the received leakage signals. In this paper, a “near-field” MFL method is proposed to quantify defect widths. Both the finite element modelling (FEM) and experimental studies are carried out to investigate the performance of the proposed method. It is found that that the distance between two peaks of the “near-field” MFL is strongly related to the defect width and lift-off value, whereas it is slightly affected by the defect depth. Based on this phenomenon, a defect width assessment relying on the “near-field” MFL method is proposed. Results show that relative judging errors are less than 5%. In addition, the analytical expression of the “near-field” MFL is also developed.

## 1. Introduction

Magnetic flux leakage (MFL) testing has been used as a high-speed and high-sensitivity non-destructive testing (NDT) method for decades [1,2,3,4]. The MFL testing has gained good performance in the detection of defects in ferromagnetic objects such as pipelines, wire ropes, tanks, etc. [5,6,7]. In practical NDT applications, qualitative and quantitative assessment methods are two of the most widely discussed problems. The qualitative assessment method gives defect information as a qualitative description, while the quantitative assessment describes the defect with quantity description such as the defect sizing and angles. In general, defect parameters are of key importance to evaluate the reliability and residual life of parts. Hence, quantitative assessments of defects by the MFL method have gained considerable attention. In the quantitative MFL method, extensive studies focus on quantifying defect parameters, i.e., depth, width, length and orientation, etc. [8,9,10].

To obtain defect parameters, the forward model plays a crucial role in the MFL method. Here, three primary MFL response-predicting methods have been established as the forward models, which involve numerical, machine learning, and analytical methods [11,12,13]. As a widely applied numerical method, the finite-element method (FEM) has been adopted to evaluate defect parameters since the 1980s [14,15,16]. The FEM is conducted by dividing the MFL model into a large number of meshes. Then, leakage magnetic field distributions are obtained through the computer. The FEM method has advantages concerning the assessment of complex defects for accuracy of calculation, the results of which are highly improved by finer meshes. However, the physical meaning behind the FEM is still missing. Consequently, a reestablished FEM is needed if any parameters are changed and a considerable amount of computation workload would occur. Hence, the FEM has achieved considerable performance with the development of computer technology. Nevertheless, it is time-consuming as a substantial amount of calculation is essential for a complex model. In the MFL, another quantitative assessment approach is the machine learning method, e.g., the neural network method [17,18,19,20,21,22,23]. The neural network method has become a hot approach in the MFL method in recent years as a primary tool for regularized direct inversion. To establish the neural network, a significant amount of training work should be completed before conducting this method. Then, judging accuracy can be highly improved by optimization algorithms. However, the biggest drawback of the neural network method concerns a tremendous amount of training data that should be obtained. Otherwise, judging accuracy would increase significantly when the training workload is very small. However, for some important industrial parts, the sample amount is too small to conduct the training work. To reduce the sample amount of the FEM and the machining learning method, the most effective forward model is the magnetic dipole method (MDM) in the MFL method [24]. In this method, the leakage magnetic field caused by a rectangle defect is the classical model. Here, a key hypothesis is proposed that the defect wall is filled with the magnetic dipole. Then, the leakage magnetic field is obtained by theoretical equations. Furthermore, the physical meaning behind this model is clear and novel algorithms can be established from this method [25,26]. In this paper, the MDM is used to evaluate the leakage magnetic fields caused by rectangle defects.

The MDM has been used in the MFL for tens of years. When the testing point is far from the defect, defect walls can be simplified as two points. Zatsepin and Shcherbinin (Z–S) have derived expressions for MFL applications when the testing point is far from defect [24,27]. For when the testing point is far from the defect, the achieved expression is the “far-field” MFL expression. The leakage will decrease sharply while increasing the lift-off values. Thus, this model has difficulty in general industrial applications. Then, Edwards and Palmer (E–P) presented an analytical solution for the leakage field of a surface-breaking crack as a function of the applied magnetic field strength, permeability, and crack dimensions. Here, the magnetic dipole density remains unchanged on defect walls [28,29]. This method provides an accurate expression when the lift-off value is not so small and this is the “middle-field” MFL expression. In fact, magnetic dipole density in the tip position would increase sharply [30]. This means the inconsistency of the magnetic charge density cannot be ignored when the lift-off value is very small, which will lead to a rough result of the analytical MFL expression. To solve this problem, the liner magnetic dipole density is proposed for small lift-off values and calculation accuracy is improved by this method [26,30]. However, expressions of the magnetic charge distribution are so complicated that it is difficult to obtain the analytical expression of the leakage magnetic field. So far, a “near-field” MFL description for defects is still a challenge.

Previous studies have shown that the distribution of the x-component of the normalized leakage magnetic field can be described as the Lorentz shape. Different from traditional MFL testing signals, two peaks would occur when decreasing lift-off values [24,30,31,32,33,34,35]. We call it the “near-field effect” in the MFL method. Two problems, i.e., the reason for and the use of this phenomenon, are still not fully investigated. The region of this phenomenon is studied both from FEM and MDM perspectives, and the “near-field effect” is applied to quantify defect width values. The rest of this paper is organized as follows. Section 2 introduces the “near-field” effect in the MFL method. Section 3 provides the experimental platform. Section 4 discusses the testing results and presents the “near-field”-based quantitative assessment method for defects. Section 5 concludes this paper.

## 2. “Near-Field Effect” in the MFL

### 2.1. FEM Model

In this section, the “near-field effect” in the MFL is studied by the FEM and MDM. Furthermore, its application in the assessment of defect width values are proposed.

**Figure 1:**
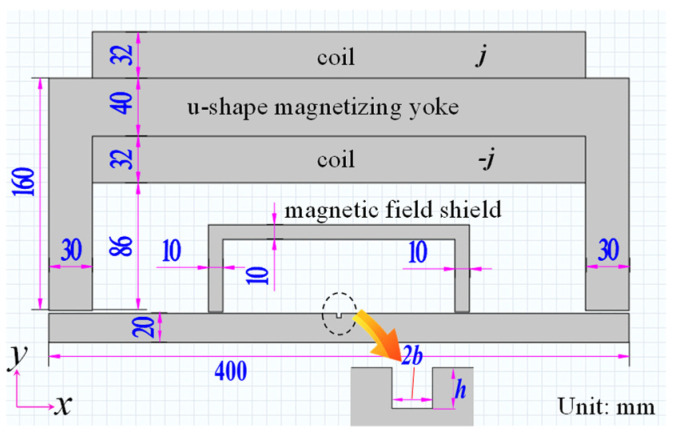
The FEM model.

The leakage magnetic field can be obtained through the MDM and FEM. In this paper, the uniform magnetic dipole distribution is of concern for a better performance in the FEM model in tiny lift-off values. To simplify the theoretical model, a 2D infinite plate is studied in COMSOL Multiphysics 4.4. A rectangle defect is in the specimen. A u-shaped magnetization yoke is applied. Then, the specimen can be the saturated magnetization state. The FEM model is illustrated in Figure 1.

**Figure 2:**
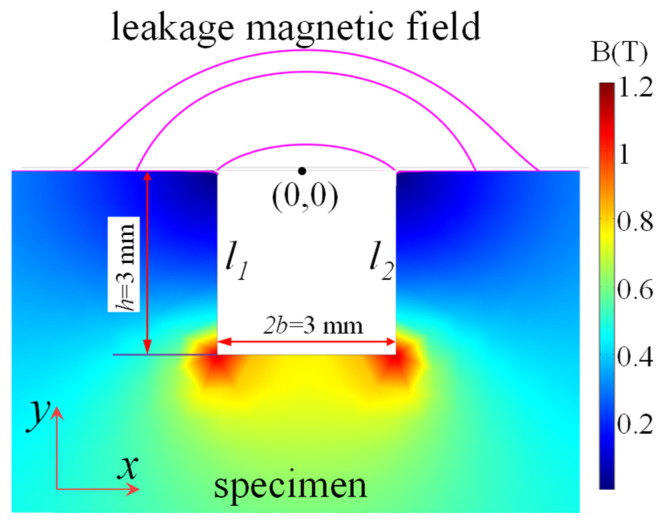
FEM result.

**Figure 3:**
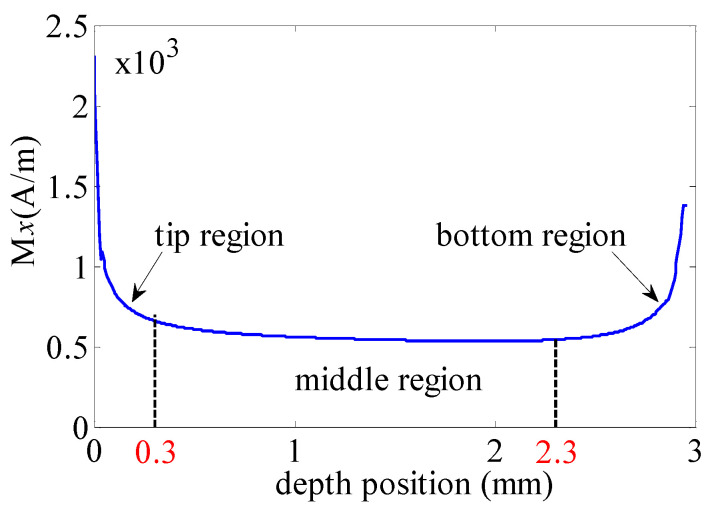
*M_x_* along the defect wall (l1).

To reduce the background magnetic field, a magnetic field shield is used. Defect parameters are 2*b* × *h* (width value × depth value). The lift-off value is *lf* (distance between testing point and specimen surface). The thickness value of the tested specimen is 20 mm. The magnetizing current density *j* is 2 × 10^5^ A/m^2^. According to the FEM analysis, when 2*b* = *h* = 3 mm, the magnetization (*M*) distribution in the defect area is shown in Figure 2. The magnetic dipole distribution (*p*(*s*)) is obtained according to Equation (1) [3,8].

(1) $$dp(s)=\vec{M}•\vec{n}dl=M_{x}dl$$

where *dp*(*s*) is the magnetic dipole. *M* is the magnetization vector and *n* is the unit vector in the x-axis direction. *S* is the depth value on the defect wall. The magnetic dipole distribution will be applied in next sections. Here, the defect wall is perpendicular to the x-axis direction. Hence, the magnetic dipole density is determined by y positions. The magnetic field in the defect region can be described as the axis-symmetric functions. Then, *M_x_* distributions in both defect walls are the same. Hence, only *M_x_* distributions in the left wall are studied. The relationship between depth position and *M_x_* is shown in Figure 3.

Here, the magnetization distribution (*H*) is divided into three regions, i.e., tip region, middle region, and bottom region. Firstly, the magnetization state changes frequently in the tip region. Then, the magnetization state is slightly changed in the middle region. Furthermore, there is also a strong variation of the magnetization state in the bottom. Then, the leakage magnetic field is calculated by Equation (2).

(2) $$\vec{H}(x,y)={\vec{H}}_{\mathrm{tip}}(x,y)+{\vec{H}}_{\mathrm{mid}}(x,y)+{\vec{H}}_{\mathrm{bot}}(x,y)$$

**Figure 4:**
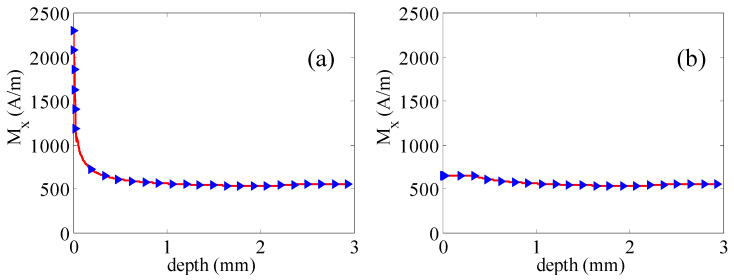
Magnetization distribution. (**a**) *M_x_* in bottom region remains unchanged and (**b**) *M_x_* remains unchanged.

Here, magnetic dipole density in the middle region is considered as an unchanged value. Then, the magnetic dipole density is shown in Figure 4a. In this situation, M_x_ in the bottom region is equal to that in the middle region, and Figure 3 is turned into Figure 4a. Comparing to traditional magnetic dipole theory, the magnetic dipole density in the wall is equal to that in the middle region as seen in Figure 4b. Then, Figure 3 is turned into Figure 4b. The leakage magnetic field can be calculated by Equation (3).

(3) $$\vec{H}(x,y)={\vec{H}}_{\mathrm{unchange}}(x,y)+f_{\mathrm{tip}}(x,y)+f_{\mathrm{bottom}}(x,y)$$

Here, *H*_unchange_(*x*,*y*) is the traditional expression of the MFL. *f*_tip_(*x*,*y*) is the deviation of the leakage magnetic field for the defect tip region and *f*_bottom_(*x*,*y*) is the deviation of the leakage magnetic field for the defect bottom region. To study the proportions of the three components, the numerical method model is established in the next section.

### 2.2. Numerical Model

**Figure 5:**
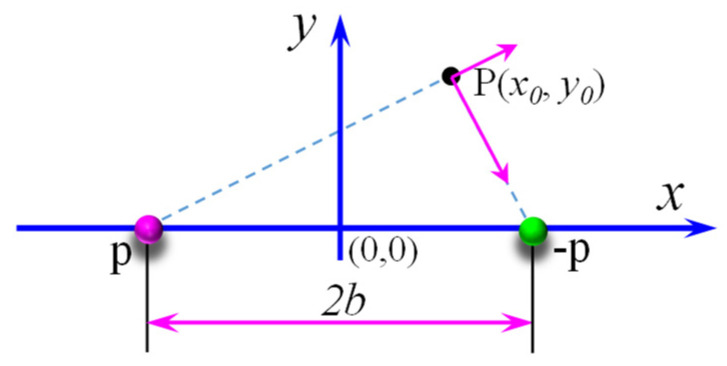
The point magnetic dipole theory.

According to the point magnetic dipole theory, the leakage magnetic field caused by the two points is shown in Figure 5 and by Equations (4) and (5).

(4) $$H_{x}(x,y)=\frac{p}{2\pi \mu_{0}}[\frac{x+b}{{\left(x+b\right)}^{2}+y^{2}}-\frac{x-b}{{\left(x-b\right)}^{2}+y^{2}}]$$

(5) $$H_{y}(x,y)=\frac{p}{2\pi \mu_{0}}[\frac{y}{{\left(x+b\right)}^{2}+y^{2}}-\frac{y}{{\left(x-b\right)}^{2}+y^{2}}]$$

Combing Equations (1), (3), and (4), the leakage magnetic field is shown as S1 in Figure 5 when the lift-off value is 1.5 mm. The leakage magnetic field is shown in Figure 5(S2) when the magnetic dipole density distribution meets Figure 4a. The leakage magnetic field is shown in Figure 5(S3) when the magnetic dipole density distribution meets Figure 4b.

**Figure 6:**
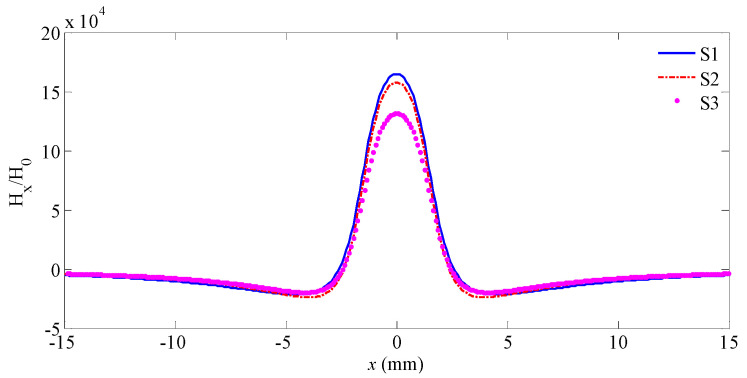
Leakage magnetic fields.

As illustrated in Figure 6, S1 is strongly similar to S2, which indicates that the leakage magnetic field is slightly affected by the non-uniform magnetic charge distribution in the bottom region. However, S3 is far different from S1. This means the leakage magnetic field is strongly affected by the non-uniform magnetic charge distribution in the tip region. Hence, Equation (3) can be turned into Equation (6).

(6) $$\vec{H}(x,y)={\vec{H}}_{\mathrm{unchange}}(x,y)+{\vec{f}}_{\mathrm{tip}}(x,y)$$

According to traditional magnetic dipole theory in the MFL, the *H*_unchange_(*x*,*y*) is shown in Equations (7) and (8).

(7) $$H_{x}(x,y)=\frac{H_{0}}{\pi_{0}}[\mathrm{atan}\frac{h(x+b)}{{\left(x+b\right)}^{2}+y(y+h)}-\mathrm{atan}\frac{h(x-b)}{{\left(x-b\right)}^{2}+y(y+h)}]$$

(8) $$H_{y}(x,y)=\frac{H_{0}}{2\pi }\ln[\frac{{\left(x+b\right)}^{2}+{\left(y+h\right)}^{2}}{{\left(x-b\right)}^{2}+{\left(y+h\right)}^{2}}\frac{{\left(x-b\right)}^{2}+y^{2}}{{\left(x+b\right)}^{2}+y^{2}}]$$

where *H*_0_ is the magnetic strength in the rectangular groove. In this paper, only the x component of the leakage magnetic field is studied. Equation (7) is transformed into Equation (9).

(9) $$H_{x}(x,y)=\frac{H_{0}}{\pi }(\mathrm{atan}\frac{x+b}{y}-\mathrm{atan}\frac{x-b}{y})-\frac{H_{0}}{\pi }(\mathrm{atan}\frac{x+b}{y+h}-\mathrm{atan}\frac{x-b}{y+h})$$

Then, Equation (6) is turned into Equation (10).

(10) $$H_{x}(x,y)=\frac{H_{0}}{\pi }(\mathrm{atan}\frac{x+b}{y}-\mathrm{atan}\frac{x-b}{y})-\frac{H_{0}}{\pi }(\mathrm{atan}\frac{x+b}{y+h}-\mathrm{atan}\frac{x-b}{y+h})+f_{x-tip}(x,y)$$

Equation (7) is turned into Equation (11) when the depth value h is an infinite number.

(11) $$H_{x}{\left(x,y\right)}_{inf\;depth}=\frac{H_{0}}{\pi }(\mathrm{atan}\frac{x+b}{y}-\mathrm{atan}\frac{x-b}{y})$$

**Figure 7:**
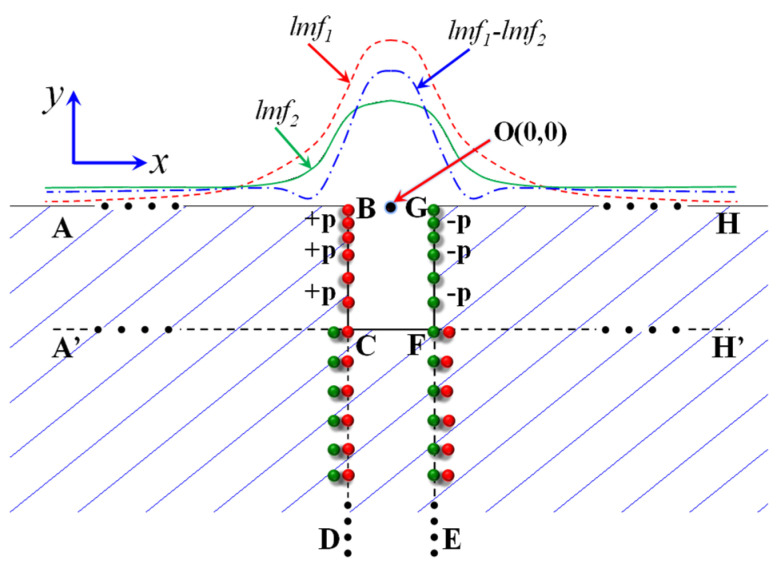
Formation mechanism of the leakage magnetic field.

Comparing with Equations (10) and (11), Equation (12) is obtained. The formation mechanism of the leakage magnetic field is shown in Figure 7. Here, *lmf*_1_ is the leakage magnetic field caused by an infinite depth crack (BDEG). The lmf2 is the leakage magnetic field caused by an infinite depth crack (CDEF). The *lmf*_1_-*lmf*_2_ is the leakage magnetic field caused by the rectangle defect (BCFG).

(12) $$H_{x}(x,y)=H_{x}{\left(x,y\right)}_{inf\;depth}-H_{x}{\left(x,y+h\right)}_{inf\;depth}+f_{x-tip}(x,y)$$

In Figure 3, the distortion of the magnetic dipole density in the tip region is slightly related to the depth value of the defect. This means the third item in Equation (12) changes the leakage magnetic fields. Conversely, the depth value h is not in the third item. Hence, the third item is only related to the width value. For the third item, the lift-off value is large and the non-uniformity of the magnetic dipole can be ignored. Here, the leakage magnetic field is calculated by the “near-field” expression as given in Equation (12). In other situations, it is worth noting that the lift-off distance cannot be ignored for low lift-off values.

### 2.3. Analytical Model

In this section, the analytical expression of “near-field” MFL is derived. As seen in Equation (12), the first and second item is achieved by the traditional magnetic dipole method. The third item is missing. In this section, leakage magnetic fields for infinite depth rectangle defects are divided. The infinite groove defect has been developed in the magnetic head for the calculation of writing the magnetic field. The leakage magnetic field is shown in Equation (13) when the “near-field effect” occurs for an infinite groove (width value is 2b).

(13) $$\begin{array}{ll} H_{x}{\left(x,y\right)}_{h\to inf} & =\frac{H_{0}}{2\pi }(\tan^{-1}(\frac{b+x}{y})+\tan^{-1}(\frac{b-x}{y}))\\ & +H_{0}\frac{b}{\sqrt{2\pi }}\left\{\frac{{\left\{\sqrt{{\left(x^{2}-y^{2}-b^{2}\right)}^{2}+4x^{2}y^{2}}-x^{2}+y^{2}+b^{2}\right\}}^{1/2}}{\sqrt{{\left(x^{2}-y^{2}-b^{2}\right)}^{2}+4y^{2}b^{2}}}\right\} \end{array}$$

The analytic expression for the “near-field effect” is obtained through Equation (14).

(14) $$\begin{array}{ll} H_{x}(x,y) & =\frac{H_{0}}{2\pi }{(\tan}^{-1}(\frac{b+x}{y})+\tan^{-1}(\frac{b-x}{y}))\\ & -\frac{H_{0}}{2\pi }{(\tan}^{-1}(\frac{b+x}{y+h})+\tan^{-1}(\frac{b-x}{y+h}))\\ & +H_{0}\frac{b}{\sqrt{2\pi }}\left\{\frac{{\left\{\sqrt{{\left(x^{2}-y^{2}-b^{2}\right)}^{2}+4x^{2}y^{2}}-x^{2}+y^{2}+b^{2}\right\}}^{1/2}}{\sqrt{{\left(x^{2}-y^{2}-b^{2}\right)}^{2}+4y^{2}b^{2}}}\right\} \end{array}$$

### 2.4. Assessment of Width Values According to the “Near-Field Effect”

**Figure 8:**
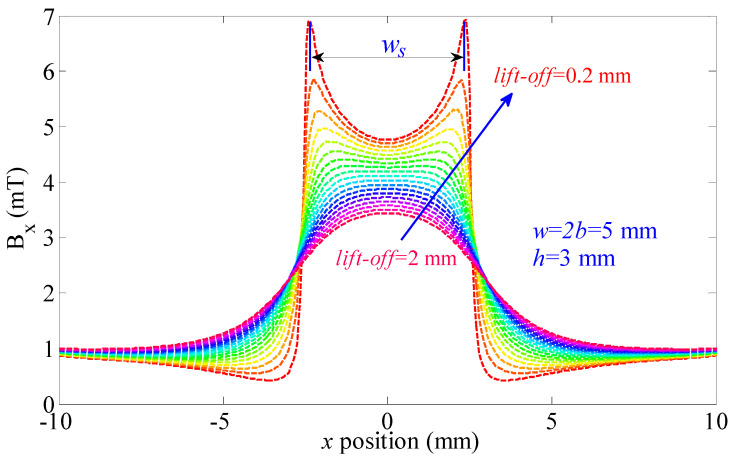
*B_x_* of the leakage magnetic field obtained in different lift-off values.

As seen in the previous section, the uniform magnetic dipole distribution in the defect tip region will change the leakage magnetic field. The distribution of the leakage magnetic field is mainly related to the width value. Hence, the “near-field effect” in the MFL can be used in the assessment of the width value. In this section, *B_x_* of the leakage magnetic field is illustrated in Figure 8 according to the FEM results (w = 5 mm, depth = 5 mm, and lift-off = 0.2~2 mm).

The typical waveform of Bx is a single-peak curve when the lift-off value is larger than 1.2 mm. A double-peak curve appears when the lift-off value is smaller than 1 mm. According to Equation (12), parameters of the distortion features are strongly related to the width value. In other words, parameters of the distortion features can be used in the assessment of defect width values. In this paper, the width value of the distortion region (*w_s_*) is studied as seen in Figure 8.

**Figure 9:**
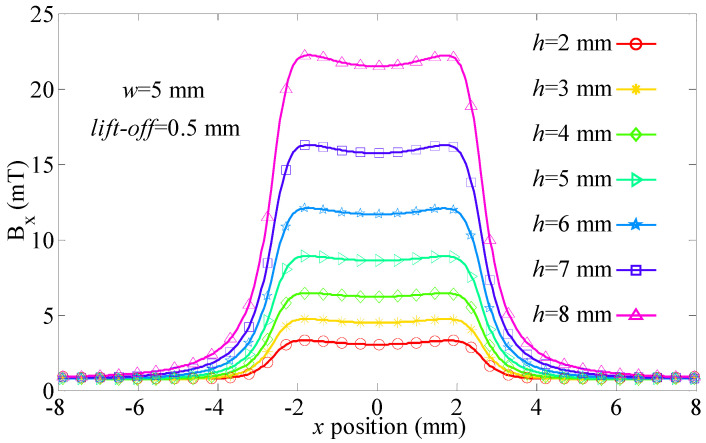
*B_x_* of the leakage magnetic field (*h* = 2~8 mm).

In other situations, *B_x_* of the leakage magnetic field is depicted in Figure 9 (lift-off = 5 mm and depth value = 2~8 mm).

Peak distance *w_s_* in Figure 9 is shown in Table 1.

As seen in Table 1, the maximum value of *w_s_* is 5.05 mm. The minimum value of *w_s_* is 4.92 mm. The relative variation of *w_s_* is *δ* = [max(*w_s_*) − in(*w_s_*)]/max(*w_s_*) × 100% = (5.05 − 4.92)/5.09 × 100% = 2.57%. Results show that *w_s_* is slightly related to defect depth value. This means the “near-field effect” has advantages in the evaluation of defect width values. In other situations, values of *w_s_* are shown in Table 2.

As seen in Table 2, δ is smaller than 5% when lift-off values are smaller than 0.7 mm. It means *w_s_* is slightly related to defect depth values in these situations. In a word, *w_s_* can be applied to evaluate the defect width value when the lift-off value is less than 0.7 mm. The values of *w_s_* are mostly due to defect width values and the evaluation algorithm has a slight relationship with defect depth values.

**Figure 10:**
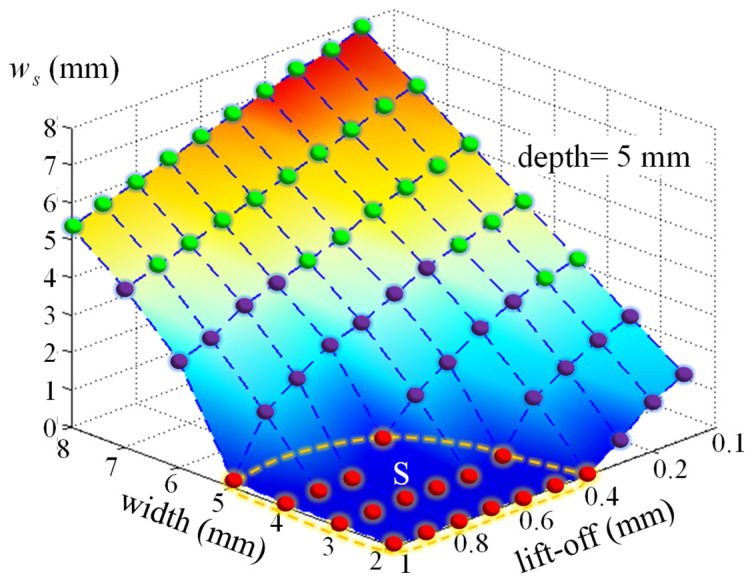
Values of *w_s_* (depth value = 5 mm).

As displayed in the aforementioned context, the “near-field effect” in the MFL can be used to assess width values. Then, we will establish the “near-field”-based width value evaluation algorithm in the MFL method. In other situations, values of *w_s_* are presented in Figure 10 when lift-off values change from 0.1 mm to 1 mm, the depth value of the defect is 5 mm, and width values change from 2 mm to 8 mm.

As illustrated in Figure 10, values of *w_s_* are zero in region S. In these extreme cases, the “near-field effect” disappears when life-off values are large and defects are small. In other cases, the “near-field effect” appears. The values of *w_s_* will increase with the increasing of width values for each lift-off value. Conversely, values of *w_s_* will increase with the increasing of larger lift-off values for each value. This means that values of *w_s_* are determined by lift-off values and defect width values. In industrial MFL applications, the lift-off value is the distance between the probe and the tested surface, and it should be the same value. Conversely, the lift-off value can be obtained if a displacement sensor is applied. Then, values of *w_s_* can be applied to deduce defect width values if the lift-off value is obtained. To establish the assessment algorithm of the defect width value by the “near-field effect”, experimental verification is conducted in the next section.

## 3. Experimental Setup

**Figure 11:**
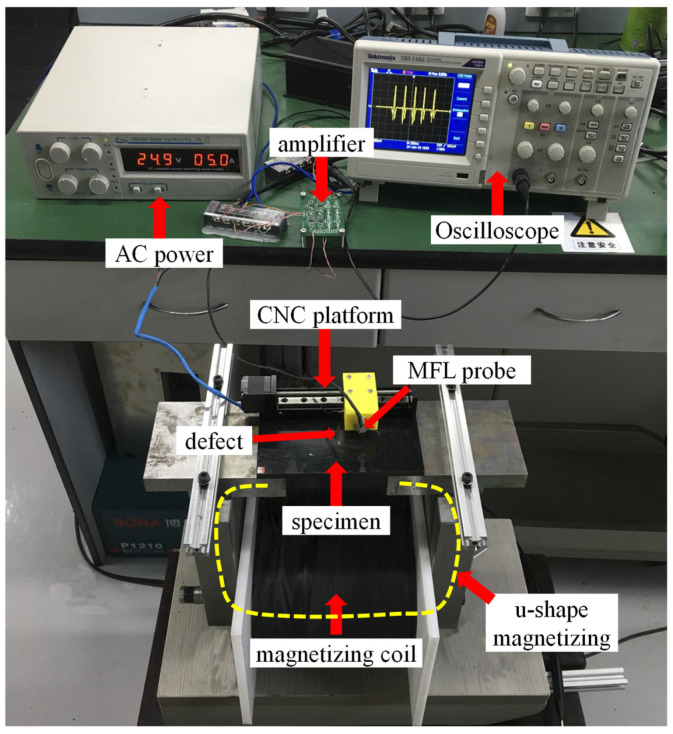
Experimental platform.

The experimental setup is given in this section as seen in Figure 11. A magnetizing coil with the turn number of 1000 is applied in the u-shape magnetizing unit. The current in the magnetizing coil is generated by the AC power. To have the specimen in the saturated magnetizing state, a DC current of 5A is applied. The MFL probe is moved by the CNC platform with a speed of 5 mm/s. The moving direction is perpendicular to the defect. Ten specimens are prepared and the thickness values are all equal to 8 mm. Rectangle defects are all in the center of each specimen with sizes of h = 5 mm and w = 3~8 mm, and w = 5 mm and h = 2~6 mm. The MFL probe is TMR2901 with a high sensitivity of 25 mV/V/Oe and the x-component of the leakage magnetic field is measured. The testing signal is processed by the amplifier with a magnification of 20 dB. MFL signals are obtained by the oscilloscope (Tektronix TBS1102) with a sampling rate of 10 MHz.

MFL signals are obtained when moving the MFL probe in different lift-off values and details of MFL signals will be presented in the next sections.

## 4. Results and Discussion

### 4.1. Studies of the “Near-Field Effect” in the MFL

**Figure 12:**
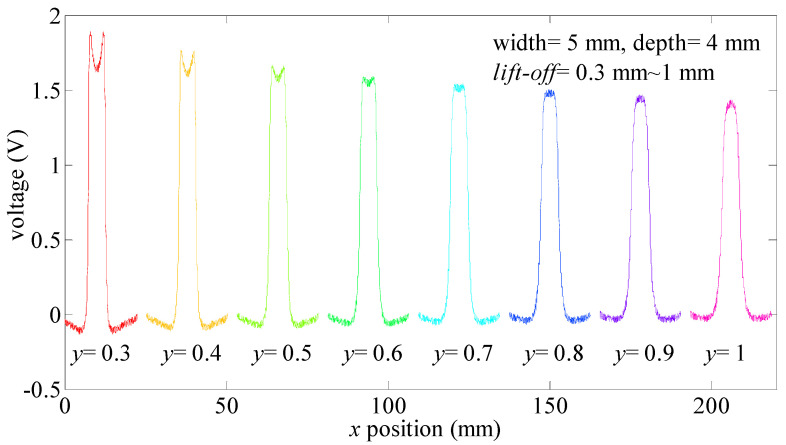
MFL signals obtained in different lift-off values.

In this section, the “near-field effect” is studied first. One defect with the size of 5 mm × 4 mm × 30 mm (width × depth × length) is tested. According to package sizes of the TMR2901, the least lift-off value is 0.3 mm. In the experimental section, MFL signals are obtained when lift-off values range from 0.3 mm to 1 mm. Testing signals are provided in Figure 12.

In Figure 12, the MFL signal turns into the bimodal signal when the lift-off value is less than 0.6 mm. The signal voltage between the maximum value and peak voltage value in the distortion region will increase while the lift-off values decrease. *w_s_* will increase while the lift-off value decreases. This means the “near-field effect” will occur with a small lift-off value compared to defect sizes. In these situations, the “near-field effect” can be applied in the assessment of defect width values.

### 4.2. Relationship between Defect Depth Values and w_s_

According to the FEM results in Section 2, the *w_s_* is slightly related to defect depth values. In this section, this conclusion is verified by experiments. Defect width values are all 5 mm. Depth values range from 2 mm to 6 mm. Lift-off values are 0.3 mm.

**Figure 13:**
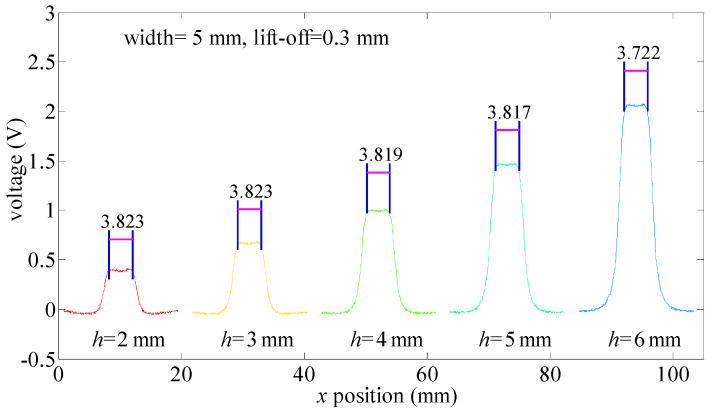
MFL testing signals for different depth values.

As seen in Figure 13, *w_s_* ranges from 3.722 mm to 3.823 mm when defect depth values change from 6 mm to 2 mm. The maximum value of *w_s_* is 3.823 mm when the defect depth value is 2 mm. The minimum value of *w_s_* is 3.722 mm when the defect depth value is 6 mm. The relative error (δ) of *w_s_* is calculated by Equation (15).

(15) $$\lambda =\frac{max(w_{s})-mim(w_{s})}{max(w_{s})}\times 100\%=\frac{3.823-3.722}{3.823}=2.75\%$$

**Figure 14:**
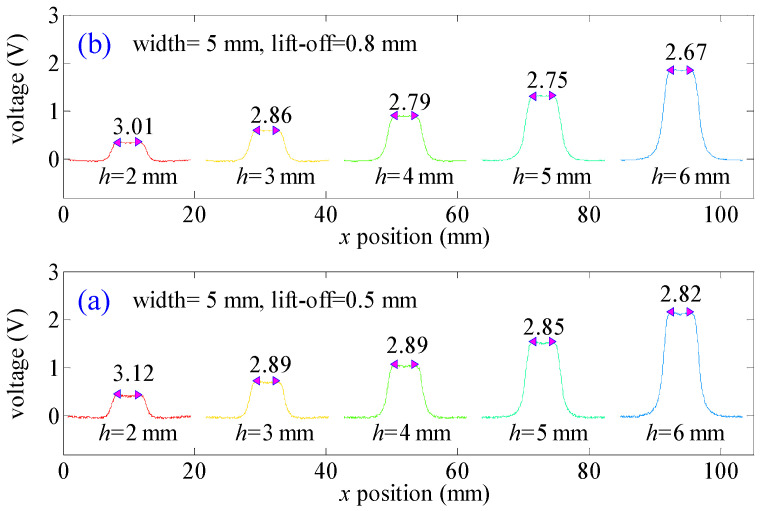
MFL signals for defects of different depth values: (**a**) lift-off value is 0.5 mm and (**b**) lift-off value is 0.8 mm.

The relative error of *w_s_* is less than 3%, indicating that the values of *w_s_* are slightly related to defect depth values when the defect width value is 5 mm and the lift-off value is 0.3 mm. In this situation, the “near-field effect” can be used to calculate defect width values. To further verify this result, experiments are conducted when the lift-off value is 0.5 mm and 0.8 mm. Testing signals are presented in Figure 14.

In Figure 14a, the relative error of *w_s_* is (3.12−2.82)/3.12 × 100% = 9.6%. In Figure 14b, the relative error of *w_s_* is (3.01−2.67)/3.01 × 100% = 11.3%. All these relative errors of *w_s_* are smaller than 12%. Experimental verification further proves that *w_s_* is slightly related to defect depth values. Hence, *w_s_* can be applied to calculate defect width values regardless of defect depth values.

### 4.3. Relationship between w_s_, Defect Width, and Lift-off Values

**Figure 15:**
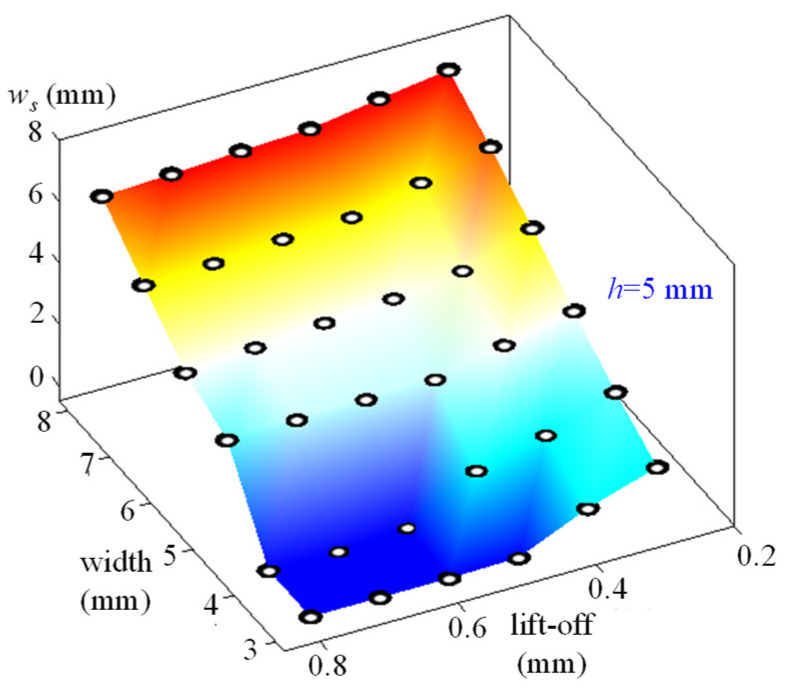
Values of *w_s_*.

In the last section, experimental verification shows that values of *w_s_* are mainly determined by defect width values and lift-off values. In this section, the relationship between *w_s_*, lift-off values, and defect width values is validated through experiments. The values of *w_s_* are shown in Figure 15.

As given in Figure 15, values of *w_s_* will increase with the increasing of width values. In addition, values of *w_s_* will increase while the lift-off values decrease. Experimental results validate the FEM results in Figure 9. To establish a quantitative relationship between values of *w_s_* and the testing parameters, the least-squares method is used in Equation (16) when *w_s_* is larger than zero.

(16) $$w_{s}=1.16\times width-2.5\times lift-1.46$$

The coefficient of determination R2 of Equation (16) is 0.97. This means the linear fitting method is appropriate to calculate *w_s_*.

According to Equation (14), the relationship between *w_s_*, width values, and lift-off values is expressed in Equation (17).

(17) $$w_{s}=1.38\times width-2.31\times lift-1.18$$

The coefficient of determination R2 of Equation (17) is 0.92. This means Equation (17) is suitable to describe the relationship between *w_s_*, lift-off values, and width values. In comparison to Equation (14), values of *w_s_* obtained by experimental verification and the magnetic dipole method will increase with the increasing of width values. Coefficients of the width value (*c_w_*) are 1.16 and 1.48. The values of *w_s_* will decrease while the lift-off values increase. Coefficients (*c_l_*) before the lift-off value are −2.5 and −2.31, respectively. Constant terms (*c_c_*) are −1.38 and −1.18, respectively. The relative error of the coefficient is determined in Equation (18).

(18) $$\delta (c)=\frac{\left|c_{1}-c_{2}\right|}{max|c_{i}|}\times 100\%$$

Then, relative errors of the coefficients are *δ*(*c_l_*) = 15.9%, *δ*(*c_c_*) = 7.6%, and *δ*(*c_w_*) = 19.2%. All relative errors for the coefficients are less than 20%. As a result, the analytic “near-field” expression is suitable to describe the leakage magnetic field. 

Equation (13) has been applied in magnetic recording technology. In these situations, the current in the recording coil is small and the relative permeability of the writing head core is very high. This means the magnetization of the head core is weak. However, the relative permeability of the tested specimen in the MFL method is not as high as the magnetic recording head cores in this paper. The analytical expression of the leakage magnetic field for saturated magnetized specimens and weakly magnetized specimens is different. Consequently, error appears when we use Equation (13) to calculate the leakage magnetic field. In future works, we will study more accurate expressions for “near-field effect” in the MFL.

### 4.4. Advantages and Disadvantages of the Proposed Method

In this paper, the “near-field effect” in the MFL method is investigated. The width values of the distortion regions are almost independent of the defect depth values, which is used to calculate defect width values in this work. This paper has proved that the linear relationship between *w_s_*, width values, and lift-off values is linear. The linear relationship can be utilized to develop the algorithm with an intuitive and low-computing cost manner.

The “near-field”-based distortion phenomenon occurs when the lift-off value is small. Hence, the proposed method can only be applied when the lift-off value is very small or the defect width value is large. In industrial applications, large-area corrosion defects always have large width values and this method can be used to calculate the width values of large-area corrosion defects. Conversely, the lift-off value is essential for the proposed method. A protective sheet with a stable thickness must be placed before the probe. During the testing process, the probe should touch the tested surface tightly. However, the thickness of the protective sheet will be reduced after a long time of operation. As a result, the proposed algorithm needs to be calibrated. In this paper, we studied basic mechanisms of the MFL method and found that all defects are rectangle defects. However, the natural defect shape is so complicated that we will study the “near-field effect” for natural defects in the future.

## 5. Conclusions

In this paper, a “near-field effect”-based MFL method is studied. A bimodal signal will appear with a close lift-off value. The bimodal distance is proved to be an independent parameter of the defect depth values and is used to quantify defect width values. The relationship between the bimodal distance, defect width value, and lift-off value is expressed as *w_s_* = 1.16 × width-2.5 × lift-1.46. The coefficient of determination *R*_2_ is 0.97. The “near-field effect” in the MFL method has advantages to calculate width values of large defects such as corrosion and indentation defects. Additionally, the analytical expression of the leakage magnetic field is developed in this paper. All relative errors for coefficients are less than 20%.

## Figures and Tables

**Table 1:** *w_s_* values in Figure 8.

*h* (mm)	2	3	4	5	6	7	8
*w_s_* (mm)	5.05	4.98	4.92	4.92	4.92	4.92	4.92

**Table 2:** Values of *w_s_* (width = 5 mm, lift-off = 0.1~1 mm, and depth = 2~8 mm).

*w_s_* (mm)							
Lift-off (mm)	Depth (mm)						δ
	2	3	5	6	7	8	
0.1	4.77	4.77	4.77	4.77	4.77	4.77	-
0.2	5.02	5.02	5.02	5.02	5.02	5.02	-
0.3	5.17	5.17	5.17	5.17	5.17	5.17	-
0.4	5.17	5.17	5.15	5.15	5.15	5.15	0.39%
0.5	5.05	4.98	4.92	4.92	4.92	4.92	2.57%
0.6	4.92	4.79	4.79	4.73	4.69	4.69	4.90%
0.7	4.79	4.67	4.54	4.41	4.41	4.41	7.93%
0.8	4.67	4.41	4.16	4.03	4.03	4.03	13.70%
0.9	4.41	4.14	3.50	3.42	3.42	3.42	22.45%
1	4.29	3.46	3.42	3.42	3.42	3.42	20.28%

## Data Availability

Not applicable.
